# 1214. Household Transmission of Febrile Illness Measured by Smartphone-Connected Thermometers, United States, 2016-2021

**DOI:** 10.1093/ofid/ofab466.1406

**Published:** 2021-12-04

**Authors:** Danielle Bloch, John Zicker, Hannah Somhegyi, Patrick Philips, Inder Singh, Amy Daitch

**Affiliations:** Kinsa Health, San Francisco, California

## Abstract

**Background:**

Understanding household transmission dynamics of infectious diseases can help develop mitigation strategies. Traditional methods of population-level disease surveillance do not capture household transmission. Data collected from smartphone-connected thermometers that can differentiate among individuals in a household can be used to study these characteristics. Using this technology, we estimated the household secondary attack rate (SAR) of febrile illness, assessed its correlation with CDC-reported influenza-like illness (ILI) and COVID-19 case incidence, and identified risk factors for secondary transmission.

**Methods:**

We conducted a retrospective cohort study among 596,096 febrile illness index cases recorded from August 1, 2016 to January 20, 2021 in households with two or more individuals in all 50 states. Fevers were measured using the Kinsa Smart Thermometer and mobile device app. Secondary cases were defined as household members who recorded a fever 1–10 days after an index case. We calculated SAR prior to and during the COVID-19 pandemic within the study period, and assessed correlation to ILI and COVID-19 case incidence using Spearman’s rank correlation coefficient. Bivariate and multivariable mixed logistic regression models were used to identify risk factors for secondary transmission.

**Results:**

SAR in the pre-COVID-19 period was 5.9% (95% CI: 5.8%–6.0%) during flu season (November to April), and 3.7% (95% CI: 3.6%–3.7%) in flu off-season, and weekly SAR was significantly correlated with ILI reported from CDC (ρ=0.84, p< 0.001). Secondary transmission was 40% more likely to occur in households where the index case’s initial temperature was ≥ 39.1°C. During the COVID-19 period, SAR was 3.3% (95% CI: 3.3%–3.4%), and daily SAR was significantly correlated with national daily COVID-19 incidence rates (ρ=0.86, p< 0.001). Households in census tracts with >50% essential workforce were 50% more likely to experience secondary transmission.

**Conclusion:**

Household SAR was highly correlated with ILI and COVID-19 cases. Capturing household transmission of febrile illness through routine public health surveillance may identify risk factors for infectious disease transmission, allowing for more targeted interventions.

**Disclosures:**

**Danielle Bloch, MPH**, **Kinsa Health** (Employee, Shareholder) **John Zicker, MS**, **Kinsa Health** (Employee, Shareholder) **Hannah Somhegyi, PhD**, **Kinsa Health** (Employee, Shareholder) **Patrick Philips, n/a**, **Kinsa Health** (Employee, Shareholder) **Inder Singh, n/a**, **Kinsa Health** (Board Member, Employee, Shareholder) **Amy Daitch, PhD**, **Kinsa Health** (Employee, Shareholder)

